# Effects of sickness manipulation on disgust and pleasantness in interpersonal touch

**DOI:** 10.1007/s00426-022-01742-3

**Published:** 2022-09-20

**Authors:** Anne Gruhl, Supreet Saluja, Richard Stevenson, Ilona Croy

**Affiliations:** 1 Department of Psychotherapy and Psychosomatic Medicine, Technische Universität Dresden, Dresden, Germany; 2 Department of Psychology, Macquarie University, Sydney, Australia; 3 Department of Clinical Psychology, Friedrich-Schiller-University Jena, Jena, Germany

## Abstract

The theory of the behavioral immune system (BIS) describes a set of behaviors that protect the individual from infectious diseases and that are motivated by disgust and the perceived vulnerability to disease. As interpersonal touch is one of the most common situations of potential transmission of infectious diseases in our everyday life, it seems likely that being touched by an apparently sick individual activates disgust. Our aim was to determine if risk of contamination from interpersonal touch alters the pleasantness of interpersonal touch and modulates facially expressed emotions. In total, 64 participants received interpersonal stroking by either a healthy or by sick-appearing experimenter. Half the strokes were performed at a slow velocity of 3 cm/s and half at a faster velocity of 30 cm/s, to modulate the degree of C-tactile fiber activation in the touch perceiver. While the experimental sickness manipulation did not influence the reported touch pleasantness, there was a tendency for a diminished expression of happiness in the slow stroking condition. In addition, the desire to clean the arm after stroking correlated positively to disgust sensitivity and to germ aversion, which is a subscale of the perceived vulnerability to disease. Contrary to previous studies, participants did not prefer the slow over the fast stroking velocity, irrespective of sickness induction. Our results lead us to assume that disgust in interpersonal touch depends especially on the touch receiver and we speculate that a rather conservative reactivity of the BIS allows for an adaptive behavioral balance in interpersonal relations. This balance may be needed to weight the risks of contamination against the benefits of interpersonal touch for social interaction.

**Supplementary Information:**

The online version contains supplementary material available at 10.1007/s00426-022-01742-3.

## Introduction

Infectious diseases are often transmitted by touching a contaminated person or object. One challenge humans have faced in their evolution is balancing the positive value of interpersonal touch—e.g., its role in bonding, intimacy, and communication—against the potential risks of pathogen spread it also carries. It has been theorized that the behavioral immune system (BIS) evolved as a response to this challenge (Aarøe et al., 2016)—i.e., a repertoire of behavioral strategies that minimize the likelihood of infectious diseases (Schaller & Park, 2011), such as influenza A (Brankston et al., 2007) or SARS-CoV-2 (Zhang et al., 2020) from entering the body. A current example of the use of avoidance strategies is the COVID-19 pandemic, where behavioral rules are excepted to effectively stop the spread of the pandemic. The compliance with those rules depends on multiple factors, such as information about the pandemic, age or the need for social interaction (Young & Brown, 2021). This example highlights that the BIS can be regarded as a complex system, which depends on many different factors (Tybur & Lieberman, 2016). Two key processes are, however, repeatedly reported to influence the BIS. The first is disgust (Murray et al., 2019), an emotion that is commonly elicited by objects or situations related to infection and illness (e.g., feces, death, insects or body fluids; (Curtis et al., 2004). The second is an individual’s belief to be vulnerable to harm posed by diseases—classified as their perceived vulnerability to disease (PVD). Thus, if someone sick or contaminated (i.e., undesirable) touches another person, this should lead to a reduction in the pleasantness of interpersonal touch and potentially elicit a disgust response. This supposition, also known as interpersonal contamination (Haidt et al., 1994), has received surprisingly little experimental attention, and so it remains unclear how humans behaviorally reduce interpersonal transmission of diseases. As such, the aim of this study was to assess whether the normally pleasant perception of interpersonal touch is affected by the potential risk of contamination (i.e., a sick person touching them), and modulated by disgust sensitivity and PVD.

A logical starting question to ask is, what is already known about behaviors that protect the body from pathogen entry? First, greater behavioral pathogen avoidance is shown to predict a decrease in the basal inflammation of the body—indicating that the BIS complements the role of the immune system in defending the body from disease (Gassen et al., 2018). In line with this, people show increased avoidance of social situations which increase infection risks, such as crowds (Wang & Ackerman, 2018), strangers (Aarøe et al., 2016; Lenk et al., 2019) or elderly people (Young & Brown, 2021). Second, an increase in disgust sensitivity has been found in the first trimester of pregnancy when the immune system is repressed (Fessler et al., 2005). This suggests that the activation of BIS is modifiable depending on an individual’s susceptibility to harm from disease. Third, there is a variability of the PVD in the context of recent disease. The PVD has not to be seen as a fixed personality trait, but as situation dependent. For example, PVD is more pronounced when a person has recently experienced an illness (Miller & Maner, 2011).

As all of these aforementioned studies have used self-report measures of the PVD and/or disgust sensitivity in imagined scenarios, one question left withstanding is whether the BIS translates to behavioral avoidance of disease in real interaction. One observation study tested this question, by assessing the development of the BIS in children aged 4–7. The researchers found that children avoid interaction with strangers in a play situation, and that this avoidance is less dependent on the age of the child and more related to the level of knowledge about the transmission of infectious diseases (Blacker & LoBue, 2016). Taken together, previous studies indicate that the BIS may have an important role in avoidance of disease, but leave open whether these results can also be applied to direct interpersonal touch.

Besides the lack of direct research on interpersonal touch, there is evidence that tactile stimuli can elicit disgust and motivate avoidance of disease. Several studies show that certain tactile properties, such as sticky, wet and soft textures, are perceived to indicate the presence of disease and that disgust to tactile objects increases when they are perceived to be a disease or contamination threat (Ilona Croy et al., 2013; Iwasa et al., 2020; Oum et al., 2011; Saluja & Stevenson, 2019). There is some evidence to indicate this extends to non-human primates—i.e., a recent study found that chimpanzees avoided consuming food when it was in close proximity to a contaminant (fecal matter), and also reduced contact with food when it was on a wet-soft object compared to a dry-hard one (Sarabian et al., 2017).

While it has not been directly assessed, two lines of evidence suggest that interpersonal touch may also activate the BIS. The first line of evidence comes from research on the so-called source effect of interpersonal disgust, which describes the phenomenon that humans express more disgust to people who have a high likelihood of bringing new pathogens into a group (Navarrete & Fessler, 2006). Consistent with this, humans report more interpersonal disgust to strangers than to friends (Lenk et al., 2019), to older people (Duncan & Schaller, 2009) and to unknown immigrants (Faulkner et al., 2004) and this effect is even more pronounced for those individuals who suffer from chronic disease (Faulkner et al., 2004) and for those who feel vulnerable to infectious diseases (Duncan & Schaller, 2009). These studies suggest that disgust can be activated upon anticipation of interpersonal contact with individuals who carry a disease risk—i.e., non-related or unfamiliar individuals (Haidt et al., 1994; Rozin et al., 1994). The second line of evidence suggesting the BIS is activated under interpersonal touch, comes from an affective touch study where participants were stroked on their forearm by a computer-controlled brush while different odors were presented (Croy et al., 2014). Not surprisingly, the presence of a fecal odor reduced pleasantness of affective touch in comparison to control odors. While these results suggest that the BIS can be triggered under interpersonal contact and modulate the pleasantness of touch, direct experimental studies investigating disgust and disease threat in interpersonal touch conditions are lacking (Murray et al., 2019).

Interpersonal touch can be applied in various ways, an often studies form is a stroking stimulation which targets C-tactile fibers. C-tactile fibers are a set of unmyelinated nerve fibers located on the hairy skin of humans and are optimally activated by light pressure stroking with a velocity of 1–10 cm/s (Löken et al., 2009)(Johansson & Vallbo, 1979) and a temperature of 32 °C (Ackerley et al., 2014). As such stimulation is typically a pleasant sensation and as humans use velocities suited to activate C-tactile fibers in interpersonal contact (Croy et al., 2016), it is assumed that those fibers are the peripheral neurological substrate for pleasant touch perception (Olausson et al., 2010).

Taken collectively, it seems that the level of disgust an individual experiences from interpersonal touch relates to two factors that have not been systematically addressed so far: first, is the perceived contamination risk posed by the touch sender—i.e., disgust will likely increase if the touch sender is perceived to carry a contagion- or sickness risk. Second, and on the receiver side, is an individual’s disgust sensitivity—a trait which varies considerably across the population (Tybur et al., 2018), and usually related to this is their PVD.

Therefore, the aim of the current study was to explore whether the normally pleasant perception of interpersonal touch is influenced by the potential risk of interpersonal contamination and by their trait levels of disgust sensitivity and by PVD.

To investigate this, we set up a cross-sectional design, in which participants were stroked on their forearm with velocities varying in the degree of C-tactile fiber stimulation (affective vs non-affective touch). The stroking was performed by either a healthy or an infectious appearing person. We hypothesized that (1) interpersonal touch (i.e., a stroke on the arm) is perceived as less pleasant, more disgusting and motivates greater arm-cleaning desire (i.e., disease avoidance) when performed by a sick person as compared to a healthy person and (2) unpleasantness, disgust and desire to clean the arm is positively related to an individual’s disgust sensitivity and PVD. We also aimed to explore whether stroking speed influences the touch perception und sickness induction.

## Materials and methods

### Design

The study was part of a larger experiment which aimed to assess the expression of disgust in proximate senses and preceded the other parts of the experiment. The participants were informed that the study was about emotion experience and expression in the proximate senses that is touch, smell and taste. Importantly, as this study was conducted in August–October 2019, our results are not influenced by the COVID-19 pandemic.

### Participants

The investigation was approved by the University of Dresden Medical School Ethics Committee and was performed in accordance with the Declaration of Helsinki. Sixty-four healthy people (51 female, 13 male), aged between 18 and 46 years (M = 25.14 SD = 6.11), participated. The required sample size of *n* = 62 participants was determined based on prior power calculations for covering a medium sized effect using G*Power (repeated-measures ANOVA, with 2 groups and 2 repeated-measures, intragroup correlation *r* = 0.3; α = 0.05, 1-β = 0.9, *f* = 0.25). Participant recruitment was stopped after reaching the number of 64 participants (*n* = 32 per group). The participants were mainly students from the Technical University of Dresden and got 10 Euro or Course-credit, if Psychology student. ﻿Exclusion criteria were self-reported current infectious illness like current colds or infectious diseases in the last 7 days, self-reported sensory impairments (taste, smell) and current severe mental symptomatology. The PHQ-9 (Patient-Health-Questionnaire-9) and PHQ-7 (Patient-Health-Questionnaire-7) averaged in our sample 6.4 for the PHQ-9 and 5 for the PHQ-7 and indicated no mayor mental health restrictions in our sample.

### Procedure

**Fig. 1:**
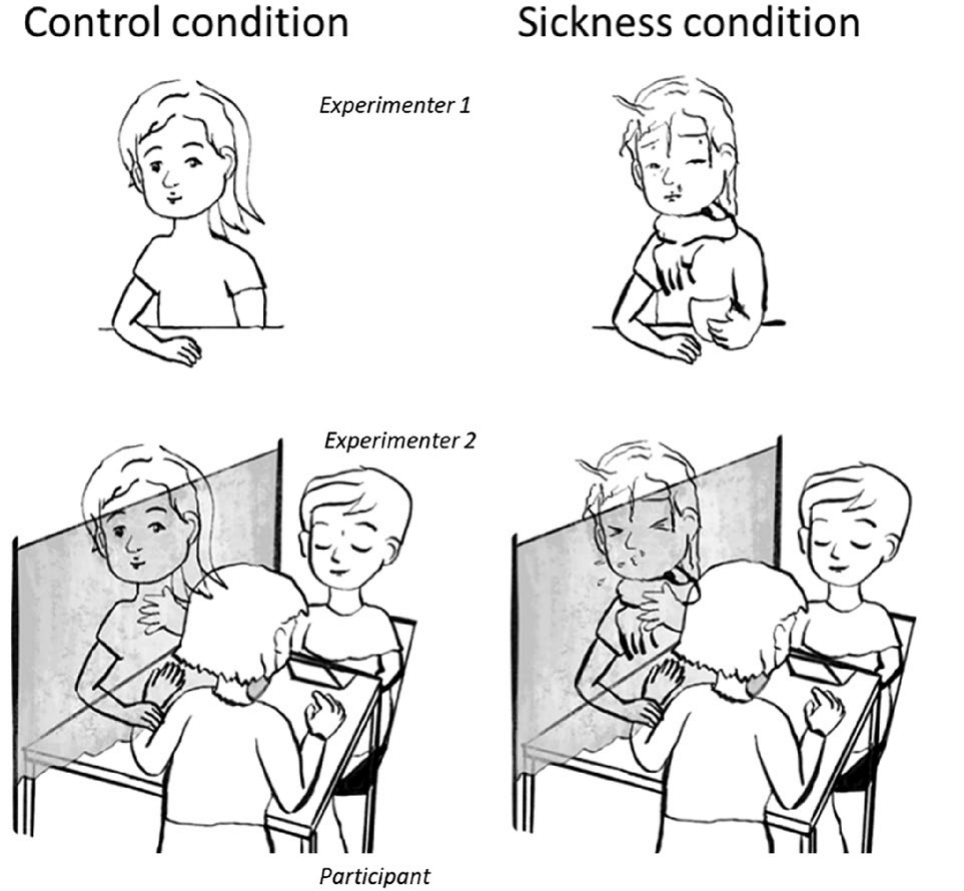
Study Design: Experimenter 1 greeted the participant and thereafter, was seated behind a paravent wall. The participant sat behind this paravent wall, and put their arm—for stroking—through an opening in the wall. Experimenter 2 was seated in front of the participant to record the ratings. A camera was positioned behind experimenter 2 to record the facial expression of the participant. In the sickness condition, experimenter 1 appeared ill; while in the control condition, she appeared healthy

The study lasted approximately 20 min and was run by two experimenters. Participants were randomly assigned to two groups with a *N* = 32 of each of them—i.e., control condition (in which the first experimenter appeared healthy) and sickness condition (in which the first experimenter appeared sick). Both groups did not differ in age or sex distribution, perceived vulnerability to disease or disgust sensitivity (Compare supplementary information 1.4). First, participants were informed about the experiment by experimenter 1 and thereafter, the experiment started. Therefore, the participant was seated behind a rectangular desk in the middle of the testing room—and the second experimenter was positioned on the other side of this desk (i.e., opposite the participant). On the left side of the participant was a black paravent wall with a small hole and a second desk behind this where the second experimenter took seat (See Fig. 1). A camera was placed in front of the participant to monitor the participants facial expression during the experiment.

The experiment consisted of three parts. In the scale-practice phase, the experimenter explained the stroke-quality scales to the participants. In the stroking-phase participants were stroked six times by the first experimenter on a 10 cm distance on their dorsal forearm. Therefore, participants placed their left arm through the hole in the paravent wall and rested it on the desk behind the wall. Any adjustments to the desk and chair position were made to ensure the participant felt relaxed. The stroking was performed without the participant being able to see it to maintain the illusion of sickness of experimenter 1 (easier fake coughing, make-up could not be examined for longer time from a short distance). The participants were informed that the blindfolded stroking helps focusing on the touch sensation. After each stroke, the participant was handed a computer with the stroke-quality scales by the second experimenter and it was not possible for experimenter 1 or 2, to see the answers of the participants as they were rating.

In the sickness condition, experimenter 1 coughed at specified points of the experiment—when the subject came into the room, while she was going behind the black wall and before the third and sixth stroke. During the experiment, participant`s facial expressions were recorded. After the stroking, participants rated the Strokers Appearance Scale, the Disgust Scale Revised and the Perceived Vulnerability to Disgust.

At the end of the larger study, participants in the sickness condition were informed about the manipulation and asked, if they believed the experimenter was sick. All participants were told about the true aims of the study, signed debrief forms and reconsented to their data being used.

### Materials and measures

Interpersonal touch stimuli. Participants were stroked by the flat hand of experimenter 1 on their dorsal forearm. We used stroking velocities of 3 cm/s for affective interpersonal touch and velocities of 30 cm/s for non-affective interpersonal touch. Each velocity was presented three times, in a randomized order. The experimenter practiced the stroking with constant force and velocity prior to the experiment with different other individuals. Furthermore, stroking was guided by a movie displaying a moving dot at the respective velocity. Experimenter 1 held her hand on a bottle filled with lukewarm water prior to and between each stroke and dried them with a towel before each stroke. This ensured that her hand temperature remained at body temperature and hands were not sweaty.

Velocities and location of the stroking stimulation were guided by research on C-tactile fibers, showing that stroking stimulation of the forearm is perceived as pleasant. C-tactile fibers are located on the hairy skin of humans and are optimally activated by light pressure stroking with a velocity of 1–10 cm/s (Löken et al., 2009)(Johansson & Vallbo, 1979) and a temperature of 32 °C (Ackerley et al., 2014). As such stimulation is typically a pleasant sensation and as humans use velocities suited to activate C-tactile fibers in interpersonal contact (Croy et al., 2016), it is assumed that those fibers are the peripheral neurological substrate for pleasant touch perception (Olausson et al., 2010).

Sickness make-up and experimental set-up. To make the stroker appear sick to the experimental group, a make-up artist was recruited and instructed the experimenter (AG) how to apply make-up so that she looked sick. This mainly constituted of increasing pallor of the skin (via applying pale foundation), adding redness around the eyes and nose (using thinly waterproof red lipstick with blue undertone) and also making the skin dewy (using skin oil). A large scarf was also worn by the stroker and she had a large teacup in her hand while explaining in the beginning of the experiment. A handkerchief pack was positioned next to her during the experiment. The sickness manipulation was confirmed as appearing significantly sicker that with normal make-up in a pre-study (compare Supplementary Materials).

Stroke-Quality Scales. The three types of scales were presented after each stroke in a randomized order on computerized-VAS lines, with a continuous slider.

*Pleasantness scale.* The pleasantness rating scale was a bipolar scale ranging from “Extremely unpleasant” ( – 50) to Extremely pleasant” (50) with neutral (0) in the middle, that indicated how pleasant the participant found each stroke.

*Emotion scales.* The participants rated how happy, scared, angry, sad, disgusted, and surprised they felt from the stoke—i.e., the six basic emotions, as defined by Ekman^27^. With the exception of disgust, all other emotions were included as distractors. Each scale ranged from “Not at all” (0) to “Very” (100).

*Cleaning desire scale*. To access the motivation for disease avoidance behavior, the participants rated their desire to clean their arm after each stroke on a range from “Not at all” (0) to “Very” (100).

Stroker appearance scales. The stroker appearance scales were only presented at the end of the experiment and all ranged from “Not at all” 0 to “Very (100).

*Health-appearance scale*. Participants were asked to rate how healthy they thought the stroker appeared. *Sickness-appearance scale*. Participants were asked to rate how sick they thought the stroker appeared. *Emotion-appearance scales*. Participants were asked to rate how (surprised, happy, sad, disgusting, angry) the stroker appeared. These scales were included as distractors.

Disgust Scale Revised (DS-R). To measure the participants individual disgust sensitivity we used The 25-item Disgust Scale Revised (DS-R; Olatunji et al., 2007). The range of this score is from 0 to 25 with a greater disgust sensitivity with higher scores. Although the Contamination subscale had low reliability in our study (α = 0.44), alphas were adequate for the other subscales (Animal-Reminder α = 0.66; Core α = 0.74); and the total score had high reliability (α = 0.79). The Contamination subscale has been shown to have a lower alpha in past research too (Olatunji et al., 2007; Overveld et al., 2006).

Perceived Vulnerability to Disease (PVD). The 15-item Perceived Vulnerability to Disease (PVD) Scale was used to measure the subject's individual risk of infection (Duncan et al., 2009). The statements were rated on a 7-point scale with a range from “strongly disagree” to “strongly agree”. The Perceived infectability subscale had adequate reliability in our study (α = 0.60), and the Germ Aversion subscale and total score had excellent reliability (> 0.84).

Patient Health Questionnaire-7 and -9 (PHQ-7 and -9). These questionnaires were used to rate symptoms of depression and anxiety (Gräfe et al., 2004)(Löwe et al., 2004).

Video evaluation. First, the six stroking sequences were clipped out of each of the videos of all subjects. These clips were then placed in a randomized order so that no assignment of stroking velocity or experimental condition was possible. The clips were saved without sound, so that no coughing was heard in the experimental condition either. Using the Mangold Interact Software, a panel code was created in order to code all occurrences of the emotions anger, disgust, happiness, sadness, fear and surprise in full length. Emotion coding was guided by the Emotion Expression Scale (see below).

Blindfolded analysis of the videos was performed by two independent video evaluators. The clips of the first 42 participants were analyzed by Evaluator A and clips from Participant 23 to 65 were analyzed by Evaluator B. The overlap of 19 participants (= 114 clips) was used for calculation of inter-rater reliability (happiness: α = 0.92; disgust: α = 0.86). In case of diverging ratings, the mean value from both evaluations was used.

Emotion coding. The gradation Emotion Expression Scale (Cohn et al., 2007) was used to evaluate the subjects' expression of happiness and disgust. This coding system provides standardized facial expression pictures which are based on Ekman’s basic emotions and allow coding of facial expressions on a 10-point scale ranging from 0 = 'no emotion' to 10 = "very extremely perceptible emotion, all facial areas maximally involved. To ensure a standardized evaluation, the evaluators were trained in advance using training videos with sample facial expressions covering different emotions and strengths, which we produced ourselves.

### Analyses

All analyses were performed using SPSS 25. Three participants were excluded from analyses as they did not believe that the stroker was healthy/sick. We tested in retrospect, whether inclusion of those participants changes the results and this was not the case. As there was no effect of time (i.e., habituation), coughing (i.e., ratings preceding the coughs were similar to those after the coughs), we averaged emotion and pleasantness over the affective and non-affective interpersonal touch stimuli, respectively. These averaged values were examined for normality using descriptive statistics (skewness and kurtosis) and Shapiro Wilks tests. Disgust- and arm-cleaning desire scores as well as facial expression data were severely skewed towards the value of zero. Hence, all analyses related to these variables were performed non-parametrically. Further, results pertaining to the distractor emotions (i.e., surprise, happiness, sad, angry, scared) are detailed in the Supplementary, as they are not of relevance to the experiment’s aims. Specific analyses related to the research questions are detailed below.

### Did the sickness induction work? 

To validate that the stroker appeared significantly more sick and less healthy to those in the sick group than in the healthy group two independent sample t-tests were run, which compared ratings on the sickness- and health-appearance scale across group. Alpha was set at 0.025 (i.e., Bonferroni adjusted; 0.05/2). Effect sizes are reported as Cohens d.

### Is pleasantness to affective or non-affective interpersonal touch reduced when the sender appears sick?

A two-way repeated-measures ANOVA on ratings of pleasantness was run, with interpersonal touch type (affective, non-affective), and group (sick vs. healthy) as factors. A Mann–Whitney *U* Test was run, comparing the facial expression of happiness as extracted from the video recording between groups.

### Is disgust and arm-cleaning desire to affective or non-affective interpersonal touch higher when the sender appears sick?

Two Wilcoxon signed rank tests were run, comparing averaged arm-cleaning desire and averaged disgust ratings between non-affective and affective touch. Alpha was Bonferroni adjusted to 0.0125 (0.05/4).

To test for the main effect of group on averaged arm-cleaning desire and disgust ratings, four Kruskal–Wallis tests were run with the between subject factor group—i.e., one for each rating (i.e., non-affective- disgust and arm-cleaning desire ratings, and affective- disgust- and arm-cleaning desire ratings). Alpha was Bonferroni adjusted to 0.0125 (0.05/4).

To test for the interaction between touch type and group, disgust and arm-cleaning desire difference scores were computed by subtracting averaged disgust and arm-cleaning desire ratings made to the affective touch from averaged ratings made to non-affective touch, respectively. Thereafter, two Kruskal–Wallis tests with the between subject factor group were run on these difference scores.

In addition, we tested whether a parametric analysis, which is typically robust against violations of normality in the given group size, reveals the same results. Those results are presented in the supplementary section.

Furthermore, a Mann–Whitney U Test was run, comparing the facial expression of disgust as extracted from the video recording between groups.

### Are scores on the disgust sensitivity and perceived vulnerability to disease related to ratings of stroking pleasantness, disgust and arm-cleaning desire?

Pearson correlations were run between total scores on the DS-R and PVD scales, their subscales and a participant’s pleasantness ratings to the affective- and non-affective touch. Spearman-rho correlations were run for analysis of disgust and desire to clean arm ratings to the affective and non-affective touch. All correlation analyses were bootstrapped (1000 iterations).

## Results

### Did the sickness induction work?

**Fig. 2:**
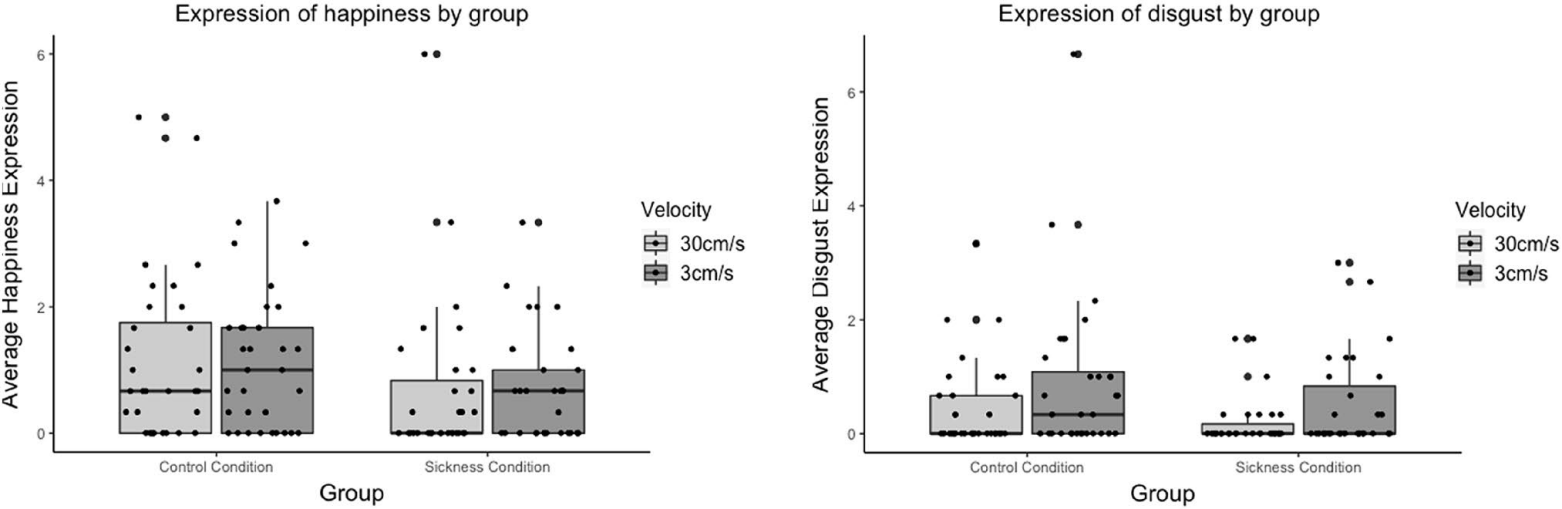
Average expression of happiness and disgust (on a scale from 0 to 10) by group and stroking velocity. There were no significant effects of sickness induction on happiness or disgust expression. Furthermore, there was no significant effect of stroking velocity and no significant interaction effect

The sickness induction worked and had a large effect size. Participants in the sick condition rated the stroker as looking significantly more sick (M = 72.3; SD = 16.4) than participants in the healthy group (M = 10.6, SD = 12.6; *p* < 0.0005; d = 4.22). Participants in the sick condition also rated the stroker as looking significantly less healthy (M = 25.2; SD = 15.3), than participants in the healthy condition (M = 77.9, SD = 17.9; *p* < 0.0005; d = 3.16) Fig. 2.

### Is pleasantness to affective or non-affective interpersonal touch reduced when the sender appears sick?

**Fig. 3:**
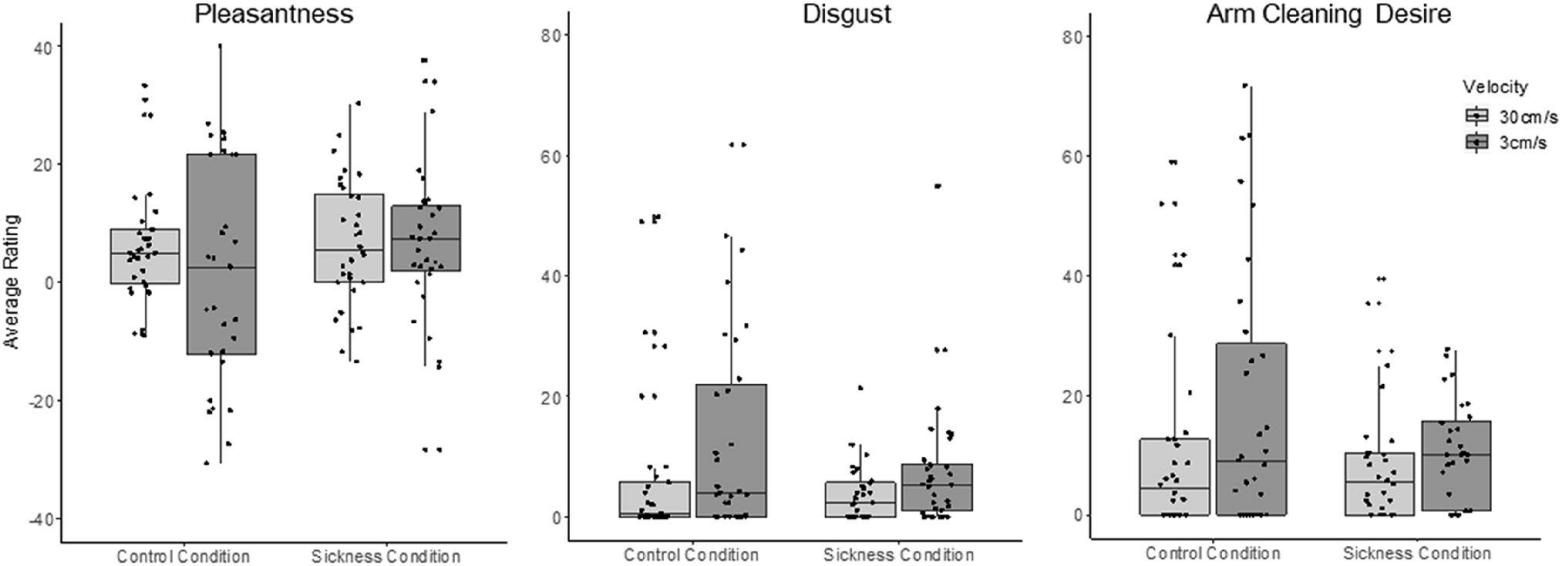
Pleasantness, disgust, and arm-cleaning desire ratings by group and stroking velocity. There were no significant effects of sickness induction on touch perception with non-parametric analysis (please see supplement for parametric analysis). Furthermore, there was no significant effect of stroking velocity and no significant interaction effect

Interpersonal touch was perceived as slightly pleasant on average and was not affected by the perceived sickness or health of the sender (Fig. 3). There was no significant main effect of interpersonal touch type *F*(1, 59) = 0.07, *p* = 0.89, η2 < 0.005, nor of group *F*(1, 59) = 0.07, *p* = 0.66, η2 = 0.002—and no interaction between these terms, *F*(1, 59) = 0.04, *p* = 0.83, η2 < 0.001.

The sickness induction reduced the facial happiness expression of the participants in the non- affective touch condition (Z = 1.51, *p* = 0.132, Fig. 2), and even more in the affective touch condition (Z = 1.94, *p* = 0.053). However, both results missed the required level of significance.

### Is disgust and arm-cleaning total desire to affective or non-affective interpersonal touch higher when the sender appears sick?

Irrespective of group and the type of interpersonal touch, disgust and arm-cleaning desire ratings were low in the sample (see Fig. 3). There was no effect of group on disgust to non-affective touch (Z = 0.57, *p* = 0.90) and affective touch (Z =  – 0.30, *p* = 1). Similarly, there was no effect of group on desire to clean arm ratings for non-affective touch (Z = 0.14 *p* = 1), and affective touch (Z = 0.09, *p* = 1). There were no significant interactions between interpersonal touch type and group for disgust ratings (Z = 0.06, *p* = 1), and arm-cleaning desire ratings (Z = 0.67, *p* = 0.82). However, a parametrical analysis revealed a significant effect of interpersonal touch type with affective touch being rated as more disgusting and provoking a higher desire to clean the arm than non-affective touch—irrespective of the group (compare Supplement 1.3).

The sickness induction did neither alter the facial disgust expression of the participants in the non- affective touch condition (Z = 1.24, *p* = 0.43, Fig. 3), nor in the affective touch condition (Z = 1.21, *p* = 0.46).

### Are scores on the disgust sensitivity and perceived vulnerability to disease related to ratings of stroking pleasantness, disgust and arm-cleaning desire?

#### Disgust sensitivity

**Fig. 4:**
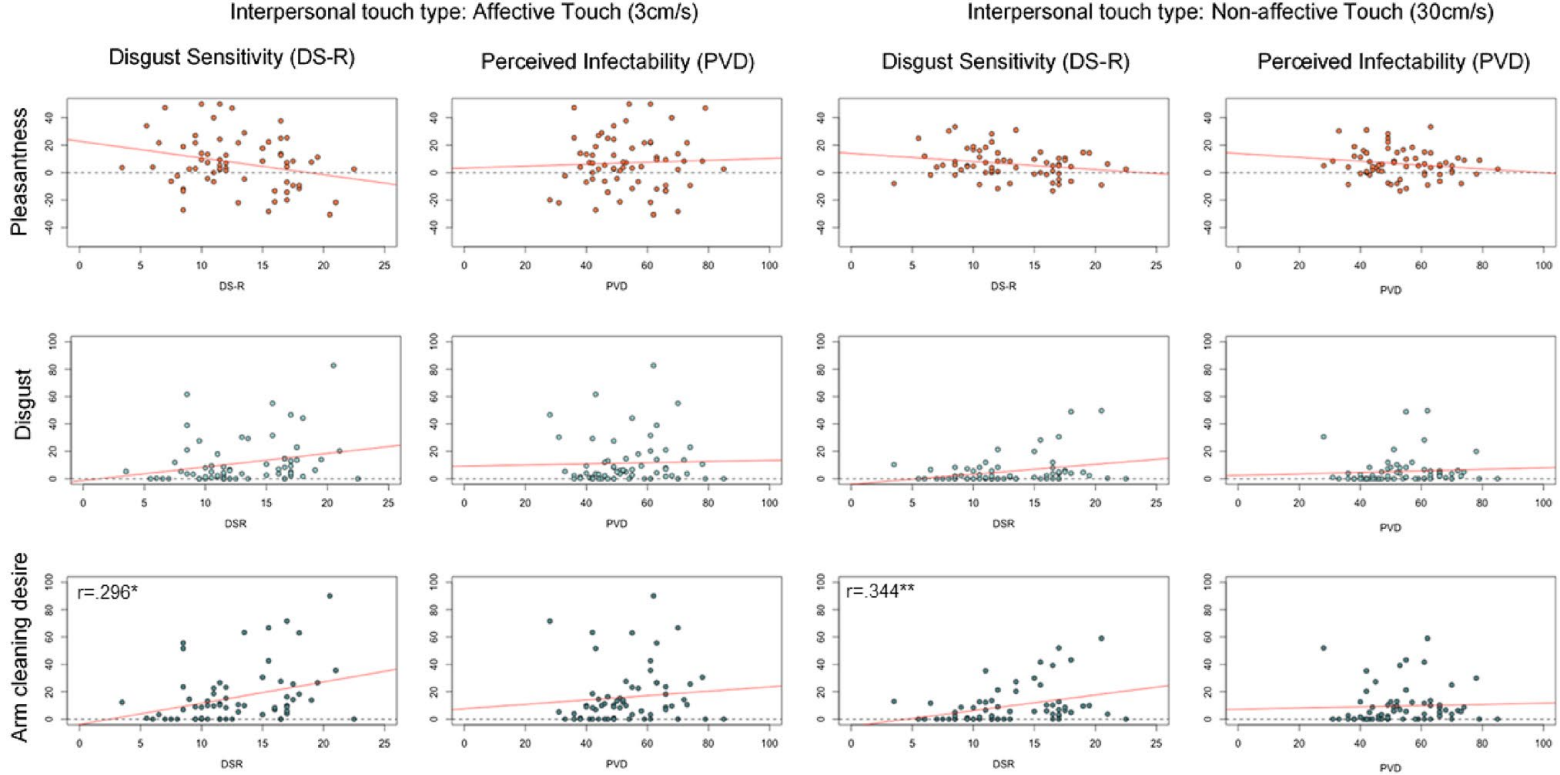
Relation between the perception of affective and non-affective stroking stimulation and disgust sensitivity (DR-R) and the perceived vulnerability to disease (PVD). R-values are displayed for significant results, for all other *r*-values, statistical threshold and confidence intervals, please compare Table 1, where DS-R equals DS-R total and PVD equals PVD total

Total DS-R negatively related to touch pleasantness and positively to arm-cleaning desire and touch disgust. However, only the correlations between DS-R and cleaning desire (both stroking velocities) reached significance (Table 1; Fig. 4). Participants with higher disgust sensitivity had a higher desire to engage in hygiene behaviors, following the interpersonal touch. Please note, that we did not correct our results for multiple testing. While such correction is effective in reducing type I error (accepting a false result), it also enhances the risk of type II errors (rejecting a true result), especially when correcting for many tests. In our study, we tested 42 potential relations between questionnaires data and stroking rating. Hence, out of chance 2.1 significant correlations can be expected. However, our data showed 7 significant correlations which speaks against an incidental finding.

**Table 1:** Correlation matrix disgust sensitivity (DS-R) and subscales, Perceived vulnerability to disease (PVD) and subscales and all interpersonal touch related ratings (30 cm/s [non-affective touch] and 3 cm/s [affective touch]

		Core disgust	Animal-reminder disgust	Contamination disgust	PVD total	Perceived infectability	Germ Aversion	Pleasantness (30 cm/s)	Pleasantness (3 cm/s)	Disgust (30 cm/s)	Disgust (3 cm/s)	Cleaning desire (30 cm/s)	Cleaning desire (3 cm/s)
*DS-R total*	*r*	,847^**^	,801^**^	,692^**^	,334^**^	*0,174*	,428^**^	*– 0,185*	*– 0,222*	*0,243*	*0,236*	,344^**^	,296^*^
	*95%CI min*	*0,764*	*0,709*	*0,535*	*0,103*	*– 0,081*	*0,184*	*– 0,401*	*– 0,456*	*– 0,019*	*– 0,028*	*0,102*	*0,051*
	*95% CI max*	*0,909*	*0,871*	*0,795*	*0,559*	*0,428*	*0,620*	*0,027*	*0,043*	*0,495*	*0,465*	*0,569*	*0,526*
*Core D*	*r*		,428^**^	,515^**^	,272^*^	*0,155*	,332^**^	– ,304^*^	*– 0,158*	*0,184*	*0,182*	,340^**^	,263^*^
	*95%CI min*		*0,216*	*0,308*	*0,057*	*– 0,073*	*0,084*	*– 0,535*	*– 0,401*	*– 0,079*	*– 0,078*	*0,103*	*0,024*
	*95% CI max*		*0,616*	*0,669*	*0,474*	*0,381*	*0,535*	*– 0,052*	*0,110*	*0,416*	*0,410*	*0,555*	*0,474*
*Animal D*	*r*			,357^**^	*0,209*	*0,071*	,318^*^	*– 0,017*	*– 0,233*	*0,245*	,257^*^	,266^*^	*0,250*
	*95%CI min*			*0,108*	*– 0,059*	*– 0,205*	*0,081*	*– 0,257*	*– 0,445*	*– 0,009*	*0,002*	*0,005*	*– 0,014*
	*95% CI max*			*0,566*	*0,467*	*0,351*	*0,535*	*0,236*	*0,031*	*0,499*	*0,479*	*0,511*	*0,488*
*Contam. D*	*r*				,364^**^	*0,237*	,405^**^	*-0,090*	*– 0,103*	*0,062*	*0,144*	*0,183*	*0,146*
	*95%CI min*				*0,102*	*-0,035*	*0,170*	*-0,299*	*– 0,328*	*– 0,221*	*– 0,139*	*– 0,075*	*– 0,130*
	*95% CI max*				*0,576*	*0,466*	*0,602*	*0,130*	*0,134*	*0,338*	*0,416*	*0,445*	*0,420*
*PVD total*	*r*					,890^**^	,802^**^	*– 0,171*	*0,071*	*0,217*	*0,079*	*0,225*	*0,210*
	*95%CI min*					*0,823*	*0,718*	*– 0,382*	*– 0,228*	*– 0,046*	*– 0,202*	*– 0,062*	*– 0,049*
	*95% CI max*					*0,938*	*0,870*	*0,054*	*0,333*	*0,441*	*0,342*	*0,464*	*0,471*
*Infectability*	*r*						,441^**^	*– 0,180*	*0,177*	*0,092*	*– 0,081*	*0,074*	*0,068*
	*95%CI min*						*0,239*	*– 0,431*	*– 0,090*	*– 0,166*	*– 0,346*	*– 0,193*	*– 0,195*
	*95% CI max*						*0,628*	*0,061*	*0,413*	*0,328*	*0,198*	*0,318*	*0,346*
*Germ Avers*	*r*							*– 0,099*	*– 0,091*	*0,251*	*0,167*	,291^*^	*0,250*
	*95%CI min*							*– 0,340*	*– 0,380*	*– 0,006*	*– 0,098*	*– 0,003*	*– 0,013*
	*95% CI max*							*0,141*	*0,191*	*0,495*	*0,421*	*0,531*	*0,473*
*Pl. 30*	*r*								*– 0,040*	– ,294^*^	*0,115*	– ,377^**^	*– 0,107*
	*95%CI min*								*– 0,271*	*– 0,529*	*– 0,152*	*– 0,591*	*– 0,368*
	*95% CI max*								*0,197*	*– 0,020*	*0,372*	*– 0,111*	*0,164*
*Pl. 3*	*r*									*– 0,169*	– ,603^**^	– ,271^*^	– ,501^**^
	*95%CI min*									*– 0,410*	*– 0,775*	*– 0,510*	*– 0,707*
	*95% CI max*									*0,096*	*– 0,398*	*– 0,005*	*– 0,253*
*D. 30*	*r*										,538^**^	,711^**^	,559^**^
	*95%CI min*										*0,288*	*0,501*	*0,305*
	*95% CI max*										*0,720*	*0,867*	*0,765*
*D 3*	*r*											,513^**^	,768^**^
	*95%CI min*											*0,247*	*0,601*
	*95% CI max*											*0,710*	*0,886*
*Clean 30*	*r*												,732^**^
	*95%CI min*												*0,533*
	*95% CI max*												*0,861*

Correlations are performed using Pearson Coefficient for Pleasantness ratings and Spearman’s Rho for Disgust and Arm-Cleaning Desire. Data are Bootstrapped and the 95% CI corridor is reported.; *p < 0.05; **p < 0.005

#### PVD

The subscale ‘germ aversion’ correlated significantly to the perceived cleaning desire (for fast velocities), all other correlations were not significant.

## Discussion

The current study showed that an individual’s disgust sensitivity and germ aversion goes along with an enhanced desire for arm-cleaning after interpersonal touch. Irrespective of how sick or healthy a touch-partner may appear, people with high disgust sensitivity and those with high germ aversion show a stronger desire to engage in disease avoidance behaviors than those with low disgust sensitivity or germ aversion. This indicates that the BIS has a role in motivating disease avoidance behaviors. In contrast to our predictions, we found no significant impact of the manipulated sickness of a touch sender on the consciously perceived pleasantness of interpersonal touch (strokes), nor on the perceived disgust or arm-cleaning desire.

Before turning to the implications of our findings, it may first be worthwhile to explain the unexpected results and limitations of the current study.

The most unexpected finding was that reported interpersonal touch pleasantness was not influenced by the sickness manipulation. This was surprising as interpersonal touch conducted by a sick individual is related to a certain risk of contamination. Hence, we would have expected BIS activation to result in decreased pleasantness of touch, increased disgust and increased motivation to clean the arm. Instead, we found a tendency for reduced expression of happiness during the sickness condition, suggesting that the risk of contamination may subconsciously influence individuals. However, this finding did not extend to the expression of disgust—an emotion which was in general expressed at a low level.

One potential shortcoming, which may explain why our sickness manipulation did not influence the perceived pleasantness nor disgust, and arm-cleaning desires, is the low ecological validity of the interpersonal touch paradigm used in this study. To get participants to focus on the stroking and remove visual bias (i.e., improve internal validity), the experimenter (who was stroking the participant’s arm) was placed behind a paravent wall, such that the participant could not see *who* was stroking them, nor their own arm*.* This differs significantly from natural interpersonal contact—in which both the touch-giver and site of touch are visible—and may have reduced participant’s belief that the touch was coming from another person. In line with this assumption, a previous study also showed smaller pleasantness differences between slow and fast stroking velocities and even unpleasant sensations, when participants were prohibited from seeing the stroking (Strauss et al., 2019). This matches our results, where also some the participants rated the stroking as unpleasant. Another point worth discussion is that, we did not observe enhanced pleasantness ratings after participants were stroked with a velocity of 3 cm/s as compared to 30 cm/s. This is in contrast to many previous studies, which report an inverted u-shaped rating pattern, with higher ratings of pleasantness for slow velocities of 3 cm/s and lower pleasantness for very slow (0.3 cm/s) and fast (30 cm/s) stroking velocities. However, at an individual level, the results of those studies show a high variability and only about 42% of healthy participants present the typical inverted u-shaped curve (Croy et al., 2021).

What we, however, did observe in the slow, as compared to the fast stroking conditions, was that disgust and arm-cleaning desire were enhanced. This fits to the idea that such stroking carries a more affective meaning (McGlone et al., 2014).

Another shortcoming was that current study used a stranger to perform an affective type of interpersonal touch—i.e., a stroke to the arm. This was done as we were interested in the modulation of interpersonal touch pleasantness. However, it is unlikely in the real world that people would be *stroked* by a stranger—and other types of touches (e.g., accidental graze, or hold) are more common (Sorokowska et al., 2021). We, therefore, assume that the more unusual-nature of the interpersonal touch experienced in this study may have resulted in lower pleasantness values and this may have superimposed a potential effect of sickness manipulation from being surfaced.

Finally, we speculate whether the sickness manipulation used in our study was severe enough to elicit a significant BIS response—as it relied on visual—i.e., pale skin, red nose and eye area, scarf, etc.—and auditory features—i.e., cough, but not olfactory factors strongly related to disgust (e.g., disease-related specific body odor; Olsson et al., 2014). Yet, given that the experimenter was rated on average as appearing very sick (i.e., most ratings were in the top quartile of the rating scale [< 75%])—it is unlikely that the sickness manipulation was not perceived as severe enough in this study to have activated some level of BIS activation. The reduced facial expression of happiness in the sickness condition may indicate that the subconscious emotion effect precedes the emotional perception.

Despite the fact that no significant BIS response was triggered by the manipulated sickness, our study showed that disgust sensitivity significantly related to arm-cleaning desire. Similarly, high germ aversion was related to a greater their desire to clean the arm. This finding is in line with previous studies reporting a significant impact of PVD on BIS reactivity (Mortensen et al., 2010)(Faulkner et al., 2004)(Duncan & Schaller, 2009). Furthermore, we replicated the correlation between DS-R and PVD. Taken together, these findings generate the hypothesis, that disgust sensitivity and PVD may enhance the perception of a potential source of contamination in interpersonal touch and this may initiate disease avoidance behavior. Future research is needed to further ascertain what roles DS-R and PVD play, in facilitating avoidance of interpersonal contact. This could for instance be studied in experimental designs with familiar and non-familiar interaction partners or in designs with even more pronounced sickness manipulation then we utilized. Observational field studies are also recommended to assess how interpersonal touch paradigms can be made more ecologically valid in experimental settings.

Considering that interpersonal touch is the most common form of daily contact—and thus a way for diseases to transmit between persons, there may be—at least in the context of interpersonal contact with unrelated individuals—a functional benefit of increased disgust sensitivity and germ aversion. In support of this, there was an increase in disgust sensitivity and germ aversion in 2019 as a result of the COVID-19 pandemic—and this increase related to greater engagement in hygiene behaviors (Stevenson et al., 2020). Hence, there may be some circumstances—e.g., pandemics, and unfamiliar contact with strangers—in which having higher trait levels of disgust sensitivity and germ aversion facilitate disease avoidance via reducing pleasant perception of touch.

In sum, we found that individual differences in disgust sensitivity and germ aversion (as part of the PVD) are related to the motivation of disease avoidance behavior, after interpersonal contact with a stranger, and this may be functional in some situations. Correlations were, however, weak to weak-to-moderate in strength. The null-finding of sickness manipulation could indicate a conservative reactivity of the BIS which may be adaptive in facilitating bonding and the role of touch in communication (Field, 2010).

## Supplementary Information

Below is the link to the electronic supplementary material.

Supplementary file1 (DOCX 178 KB)
