# Supplementary material for: Effects of sickness manipulation on disgust and pleasantness in interpersonal touch
Source: Psychol Res. 2022 Sep 20;87(5):1454–65. doi: 10.1007/s00426-022-01742-3 (PMC9489268; doi:10.1007/s00426-022-01742-3)
Supplement: Supplementary file 1 — Supplementary file1 (DOCX 178 KB) [file 426_2022_1742_MOESM1_ESM.docx]

# **Supplement**

Paper - Effects of sickness manipulation on disgust and pleasantness in interpersonal touch

## Pre-Study

To investigate the modulation of touch perception in the context of sickness manipulation we created the illusion of a potentially infectious stroker. We choose to use therefore changes in the environment (hence teacup, handkerchief) and disease associated behavioural changes (coughing; as well visual signs of the strokers illness). For the strokers visual illness appearance, we used a make-up, which consisted of pale skin, reddened eye and nose area and some oil as sweat simulation.

To ensure a well worked sickness illusion in our main experiment, we conducted a pre-study in advance to test the effect of the make-up.

### Methods

The pre-study showed a picture of the face of the stroker once with special make-up for the illusion of illness and once without, as well as two distracter images of another person without the make-up with once a happy and once a sad face expression.

The make-up mainly constituted of increasing pallor of the skin (via applying pale foundation), adding redness around the eyes and nose (using thinly waterproof red lipstick with blue undertone) and also making the skin dewy (using skin oil).

The pictures were then sent in a questionnaire to 21 participants who were asked to rate the person in the pictures. As in the main experiment, the subjects were mainly students.

The questionnaires contained characteristic scales, which were to evaluate the person on the photos. We were particularly interested in how sick or healthy the person appeared. We also added happy, sad, angry, surprised and scared as distracting characteristics.

**Appearance Scales.**

The appearance scales all ranged from “Not at all” 0 to “Very (100).

*Health-appearance scale***.** Participants were asked to rate how healthy they thought the person in the image appeared. *Sickness-appearance scale***.** Participants were asked to rate how sick they thought the person in the image. *Emotion-appearance scales*. Participants were asked to rate how (happy, sad, angry, surprised, scared) the person in the image appeared. These scales were included as distractors.

### Analysis

Two paired samples t-test were run. The first compared the sickness-appearance ratings made to photos of the stroker with sickness makeup and without sickness makeup – and similarly, the second compared the health appearance ratings, made to the stroker with and without makeup.

### Results

The stroker was rated as significantly more sick with sickness makeup than without sickness makeup (t(20) = 5.76, *p* < 0.0005), and as significantly less healthy with sickness makeup than without sickness makeup (t(20) = -5.90, *p* < 0.0005)., *p* < 0.0005). These results indicated that the stroker with sickness makeup was perceived to be significantly more skin and less healthy, than the stroker without the sickness-makeup (See Supplementary Table 1 for means and SDs)

Table S1. Pilot Appearance Rating Scale data Means and SDs

|  | **Condition** | **M (SD)** |
| --- | --- | --- |
| **Health Appearance** | Sickness-makeup | 31.86 (21.82) |
|  | No makeup | 63.24 (19.12) |
| **Sickness Appearance** | Sickness-makeup | 41.33 (28.89) |
|  | No makeup | 5.81 (6.79) |

## Distracter Emotions

We used 5 distraction emotions in our questionnaires: happy, scared, angry, sad and surprised, based on the 6 basic emotions according to Ekman (Ekman, 1992). The aim of the distractors was to ensure that the probands could not infer the exact emotion we were concerned with in the experiment.

Mean ratings and correlations of those rating are displayed below.

Table S2: Mean, SD and Correlation of the Stroke-Quality-Scales

|  | **Mean** | **SD** | Average  pleasant | Average clean arm | Average disgusting | Average happy | Average scared | Average angry | Average sad | Average  surprised |
| --- | --- | --- | --- | --- | --- | --- | --- | --- | --- | --- |
| Average pleasant | 6.714 | 11.17 |  |  |  |  |  |  |  |  |
| Average clean arm | 12.916 | 16.021 | **-0.476***** |  |  |  |  |  |  |  |
| Average disgusting | 8.464 | 12.317 | **-0.515***** | **0.870***** |  |  |  |  |  |  |
| Average happy | 38.495 | 21.554 | **0.346*** | -0.026 | -0.046 |  |  |  |  |  |
| Average scared | 5.411 | 8.600 | **-0.420***** | **0.703***** | **0.715***** | 0.029 |  |  |  |  |
| Average angry | 2.000 | 6.488 | -0.280 | **0.576***** | **0.673***** | 0.046 | **0.681***** |  |  |  |
| Average  sad | 2.669 | 7.625 | -0.252 | **0.473***** | **0.586***** | 0.084 | **0.658***** | **0.920***** |  |  |
| Average  surprised | 24.478 | 17.111 | -0.308 | **0.528***** | **0.572***** | 0.109 | **0.629***** | 0.375 | 0.364 |  |
| Signif. codes: 0 ‘***’ 0.001 ‘**’ 0.01 ‘*’ 0.05 ‘.’ 0.1 ‘ ’ 1 | | | | | | | | | | |
|  | | | | | | | | | | |


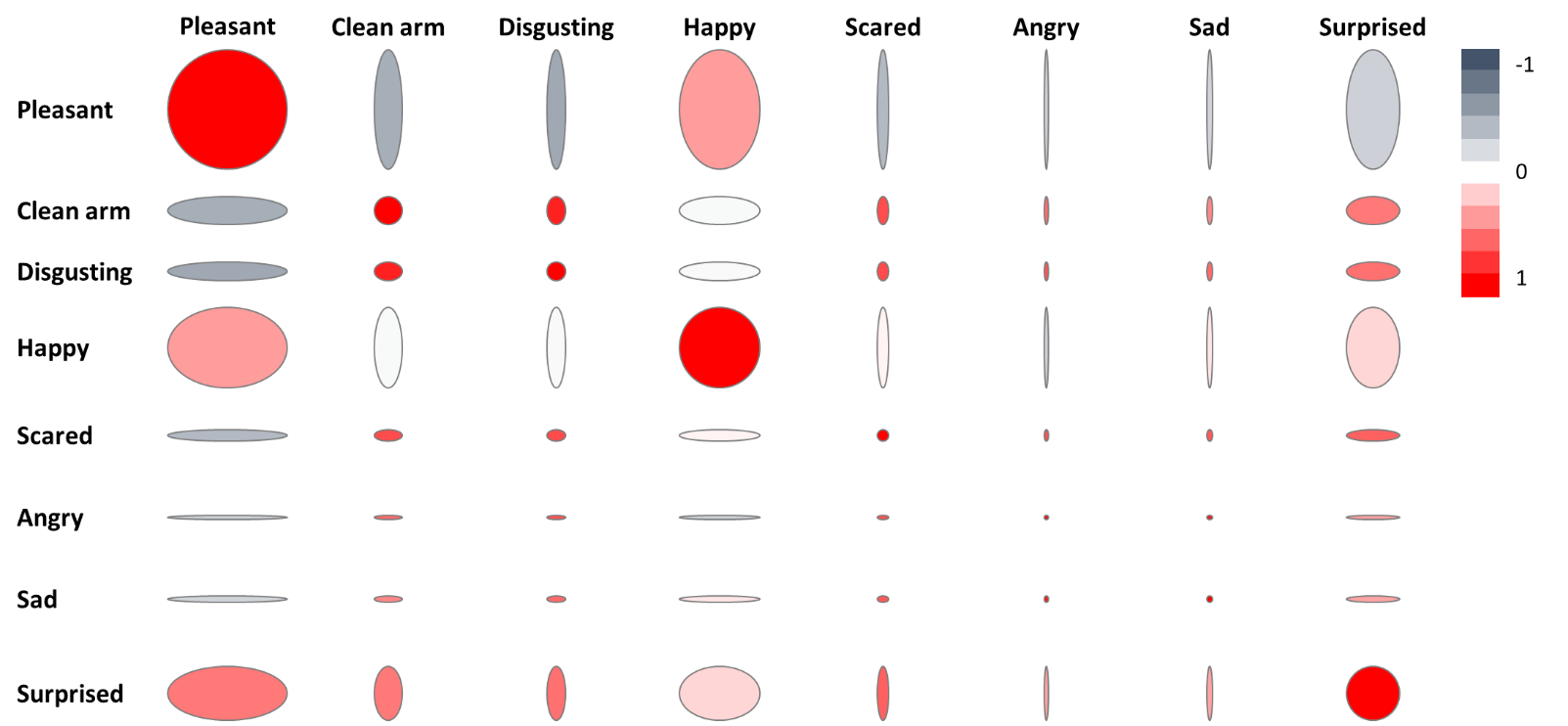


Figure S1: Visualisation of the correlation of all rating scales. Size of the bubbles indicates the average ratings, color indicates the correlation.

## Parametrical analysis of disgust and arm-cleaning

Parametrical analysis revealed a stronger disgust and arm cleaning desire for slow as compared to fast touch.

Arm-cleaning: significant main effect of interpersonal touch type F(1, 59) = 16.3, p < .001, η2 = .216, no significant effect of Group F(1, 59) = 1.7, p = .199, η2 = .028 and no significant interaction between these terms, F(1, 59) = 2.8, p = .100, η2 = .045

Disgust: significant main effect of interpersonal touch type F(1, 59) = 13.6, p < .001, η2 = . 187, no significant effect of Group F(1, 59) = 2.3, p = .133, η2 = .038 and no significant interaction between these terms, F(1, 59) = 1.4, p = .100, η2 = .024

## Descriptive statistics of participants in the control and sickness condition

The distribution of age (p=0.420), sex (p=0.536), perceived vulnerability to disease (p=0.768) and disgust sensitivity (p=0.523) was evenly between the groups. There was also no significant difference in the homogeneity of the groups.

Table S3: Descriptive statistics of participants in the Control and Sickness Condition

| Group | Control condition | Sickness condition | Control condition | Sickness condition |
| --- | --- | --- | --- | --- |
|  | Number of females | | Percentage of females | |
| Sex | 24 | 27 | 75 | 84.4 |
|  | Mean |  | Standard deviation | |
| Age | 24.44 | 4.63 | 4.63 | 7.31 |
| DS-R total | 12.8 | 13.1 | 4.16 | 4.26 |
| PVD total | 54.8 | 52.7 | 13.1 | 12.6 |

## Video Evaluation

The video evaluation is done by two independent video evaluators. After their training, they were able to demonstrate a high degree of internal consistency in the evaluation. This was shown as follows:

Table S4: Mean, SD, Cronbachs α and McDonald's ω for coding the Expression of happy Video Evaluator A and B

| Scale Reliability Statistics for coding Expression scale: happy | | | | | | | | | |
| --- | --- | --- | --- | --- | --- | --- | --- | --- | --- |
|  |  |  |  |  |  |  |  |  |  |
|  | | **mean** | | **sd** | | **Cronbach's α** | | **McDonald's ω** | |
| scale |  | 1.08 |  | 1.35 |  | 0.946 |  | 0.949 |  |
|  | | | | | | | | | |
|  | | | | | | | | | |

Table S5: Correlation of the Stroke-Quality-Scales of the Video Evaluator A and B

|  | Event 1 happy B | Event 2 happy B | Event 3 happy B | Event 4 happy B | Event 5 happy B | Event 6  happy B |
| --- | --- | --- | --- | --- | --- | --- |
| Event 1 happy A | **0,96***** |  |  |  |  |  |
| Event 2 happy A |  | **0,95***** |  |  |  |  |
| Event 3 happy A |  |  | **0,89***** |  |  |  |
| Event 4 happy A |  |  |  | **0,96***** |  |  |
| Event 5 happy A |  |  |  |  | **0,72***** |  |
| Event 6 happy A |  |  |  |  |  | **0,95***** |
| Signif. codes: 0 ‘***’ 0.001 ‘**’ 0.01 ‘*’ 0.05 ‘.’ 0.1 ‘ ’ 1 | | | | | | |

Table S6: Mean, SD, Cronbachs α and McDonald's ω for coding the Expression of disgust Video Evaluator A and B

| Scale Reliability Statistics for coding Expression scale: disgust | | | | | | | | | |
| --- | --- | --- | --- | --- | --- | --- | --- | --- | --- |
|  |  |  |  |  |  |  |  |  |  |
|  | | **mean** | | **sd** | | **Cronbach's α** | | **McDonald's ω** | |
| scale |  | 0.592 |  | 0.754 |  | 0.889 |  | 0.887 |  |

 Table S7: Correlation of the Stroke-Quality-Scales of the Video Evaluator A and B

|  | Event 1 disgust B | Event 2 disgust B | Event 3 disgust B | Event 4 disgust B | Event 5 disgust B | Event 6  disgust B |
| --- | --- | --- | --- | --- | --- | --- |
| Event 1 disgust A | **0,81***** |  |  |  |  |  |
| Event 2 disgust A |  | **0,91***** |  |  |  |  |
| Event 3 disgust A |  |  | **0,87***** |  |  |  |
| Event 4 disgust A |  |  |  | **0,89***** |  |  |
| Event 5 disgust A |  |  |  |  | **0,82***** |  |
| Event 6 disgust A |  |  |  |  |  | **0,92***** |
| Signif. codes: 0 ‘***’ 0.001 ‘**’ 0.01 ‘*’ 0.05 | | | | | | |

***Bibliography:***

Ekman, P. (1992). Are There Basic Emotions? *Psychological Review*, *99*(3), 550–553. https://doi.org/10.1037/0033-295X.99.3.550
